# Blood Lactate or Lactate Clearance: Which Is Robust to Predict the Neurological Outcomes after Cardiac Arrest? A Systematic Review and Meta-Analysis

**DOI:** 10.1155/2018/8014213

**Published:** 2018-10-02

**Authors:** Bao-Chun Zhou, Zheng Zhang, Jian-Jun Zhu, Li-Jun Liu, Chun-Feng Liu

**Affiliations:** ^1^Department of Emergency and Critical Care Medicine, The Second Affiliated Hospital of Soochow University, No. 1055, San Xiang Road, Suzhou 215004, Jiangsu, China; ^2^Department of Nutrition and Food Hygiene, School of Public Health, Soochow University, 199 Renai Road, Suzhou, Jiangsu Province 215123, China; ^3^Department of Neurology, The Second Affiliated Hospital of Soochow University, No. 1055, San Xiang Road, Suzhou 215004, Jiangsu, China

## Abstract

**Aims:**

Lactate and lactate clearance were supposed to be associated with cardiac arrest outcomes, but studies obtained different results. Thus, we conducted this meta-analysis to investigate the association between lactate or lactate clearance and neurological outcomes and their usefulness for prediction of neurological outcomes.

**Methods:**

We conducted a systematic search in PubMed, Web of science, EMBASE, Medline, and Google Scholar until May 1, 2018, for relevant studies. Studies reporting lactate, lactate clearance on admission, or other time points after admission associated with neurological outcomes were included in our analysis. Pooled effect date was shown as weighed mean difference (WMD) and 95% confidence interval (CI). To measure the usefulness of lactate on admission to predict neurological outcomes, we also pooled the data of diagnostic test.

**Results:**

23 studies involving 6720 cardiac arrest (CA) patients were included. Results from our analysis indicated that patients with good neurological outcomes tended to have a lower lactate level on admission (WMD: -2.66 mmol/L, 95%CI: -3.39 to -1.93) and 12h, 24h, and 48h after admission (*P*<0.001). Furthermore, the pooled AUC for lactate level on admission to predict neurological outcomes was 0.77 (95%CI: 0.73-0.80). However, a significant association between lactate clearance and neurological outcomes was only found in 24h but not 12h lactate clearance rate.

**Conclusions:**

Lactate levels on admission and all time points up to 48h were associated with neurological outcomes after CA, whereas the association between lactate clearance and neurological outcomes was not so stable. Lactate was a more robust surrogate marker than lactate clearance to predict neurological outcomes after CA.

## 1. Introduction

Cardiac arrest (CA) presents a serious public health concern with high mortality cause by various pathogenesis such as anoxia, drug, electrolyte disturbance, low temperature, and hypoglycemia [1]. In Europe and north American, CA causes more than 600,000 cases per year [2], and the survival rate at hospital discharge is very low. Two major reasons for the mortality after resuscitation are postresuscitation circulatory failure and postanoxic neuro injury. Many researches have performed studies to develop tools for predicting the neurological outcomes. Biological markers such as neuron-specific enolase and S-100*β* protein have been demonstrated to be efficient predictors for neurological outcomes after CA. But these markers need longer time for detection. Therefore, a facility marker to predict neurological is needed in clinical practice. Lactate and lactated clearance are supposed to be that kind of marker.

Lactate is a product of pyruvate reduction during glycolysis. During CA, tissue hypoxia leads to accumulation of pyruvate and therewith the accumulation of lactate. The level of circulation lactate is likely to be a good marker of tissue hypoxia which is also recommended by Utstein guidelines. Not only lactated level but also a lactate clearance from serial determination of lactate is supposed to associated with cardiac arrest outcomes of CA [3, 4], and a serial determination of lactate is also recommended in CA survival after return of spontaneous circulation (ROSC) [5, 6]. But studies obtain discrepant results [3, 4, 7–11]. This also leads to some confusions in the application of lactate or lactate clearance to predict neurological outcomes in clinic. In order to figure out the relationship between lactate or lactate clearance at different time points and neurological outcomes after CA and compare the efficiency of lactate or lactate clearance in predicting neurological outcomes after CA, we carry out a meta-analysis to summarize the current evidence.

## 2. Materials and Methods

### 2.1. Search Strategy

We carried out a literature search on PubMed database, Medline, EMBASE, Web of Science, and Google Scholar. We searched these databases until May 1, 2018, for studies containing the following keyword and/or Medical Subject Heading terms: (1) lactate OR hyperlactatemia OR lactate acid OR lactate clearance OR lactic clearance OR lactate normalization OR lactate kinetics, (2) heart arrest OR out of hospital cardiac arrest OR OHCA, and (3) neurological outcome OR cardiac arrest outcome OR CA outcome.

Besides, we also reviewed the reference lists of relevant articles to identify additional studies. Letters to author or abstract were scrutinized and excluded because they lacked sufficient data for analysis.

### 2.2. Study Selection

Studies were selected into our meta-analysis based on the following criteria: (1) carried on nontrauma cardiac arrest patients; (2) reported lactate level on admission or other time points after admission related to neurological outcomes; (3) reported lactate clearance related to neurological outcomes; (4) reported the detailed data of prognostic test. Studies were excluded in the following cases: (1) if it did not report lactate level related to neurological outcomes, (2) letter to author, and (3) study abstract.

### 2.3. Data Extraction

The following data were extracted from each eligible study: the first author's last name, year of publication, study design, geographic location, number of subjects, mean age of subjects, gender of subjects, percentage of initial shockable rhythm, location of CA, outcome follow-up time, therapeutic hypothermia, the sources where lactate measures from, neurological outcomes, and mean lactate level on admission. Besides, the data of serum lactate level and lactate clearance related to neurological outcomes at different time points were also extracted. To make an overview of lactate diagnostic efficiency, date of diagnostic test was also extracted. Lactate cut-off point, lactate measured time, neurological outcome measured time, and absolute value of true positive, true negative, false positive, and false negative were retrieved or developed 2x2 contingency table. Study selection and date extraction were conducted independently by two authors (Z-BC and Z-Z), with any disagreements resolved by consensus.

### 2.4. Definition of Neurological Outcome

The primary end point was neurological outcomes at discharge from hospital or another follow-up time. Most of studies used Glasgow-Pittsburgh cerebral performance categories (CPC) to measure the neurological outcome after CA [3, 10, 12–18, 20–31]. Good neurological outcome was defined as a CPC of 1-2 and poor neurological outcome was defined as a CPC of 3-5. One study employed Glasgow Coma Score (GCS) on hospital discharge to measure the neurological outcome [19]. Good neurological outcome was defined as a GCS of 14-15. Another study adopting modified Rankin scale (mRS) to measure neurological outcome and good neurological outcome was defined as a mRS of 0-3.

### 2.5. Methodological Quality Assessment

The methodological quality assessment of eligible studies was appraised using Newcastle-Ottawa Scale (NOS) [32]. Articles with scores <4, between 4 and 6, or >6 were considered as low, intermediate, or high quality, respectively. The assessment was also carried out by two authors (Z-BC and Z-Z). If there was any disagreement, a third author would reevaluate the original study.

### 2.6. Statistical Methods

Effect size of lactate difference between different neurological outcomes patients was defined as weighed mean difference (WMD) and 95% confidence interval (CI). Articles reported data as interquartile range and median [4, 10, 13–17, 19, 21, 23–25, 27, 30, 31]. We converted data into mean and s.d. according to the method developed by Wan [33]. The unit of lactate was uniformed to mmol/L. Lactate measured at initial, on the arrival of hospital, emergency department, or ICU was defined as admission lactate level. Lactate measured within one hour after admission was also defined as lactate on admission. Besides, we also analyzed lactate level at 12h, 24h, and 48h after admission related to neurological outcomes. We used the Cochran Q and I^2^ statistics to assess heterogeneity among studies. For the Q statistic, a* P* value < 0.1 was considered statistically significant heterogeneity. For the I^2^ statistics, a value greater than 50% was considered high heterogeneity [34]. We used a random-effects model to estimate WMD in case of heterogeneity. Statistical synthesis and data analysis of diagnostic test were conducted according to the method introduced by Lee [35]. Sensitivity, specificity, likelihood ratios, diagnostic odds ratios, and receiver operating characteristic curves (SROC) were pooled using the DerSimonian and Laird method (random-effects model). To analyze the threshold effect, Spearman correlation coefficient of sensitivity and 1-specificity was calculated.

Considering that the differences in trails design and baseline characteristic of patients may contribute to the obvious heterogeneity observed in our analysis, we also designed subgroup analysis and metaregression analysis to explore the vital baseline factors. We carried out subgroup analysis according to mean age (adult versus old), CA location (OHCA versus OHCA/IHCA) (OHCA: out of hospital cardiac arrest; IHCA: in hospital cardiac arrest), initial shockable rhythm (high percentage versus low percentage), quality of included studies (high versus intermediate), data transformation (Yes versus No), and outcome follow-up time (long versus short-term). Old people were defined as patients above 65 years old. Long-term follow-up was defined as measured neurological outcomes 3 months or longer after CA. Metaregression analysis carried out subgroup analysis according to mean age, percentage of initial shockable rhythm, and mean lactate level on admission.

Sensitivity analysis was employed to assess the stability of the meta-analysis. The publication bias was assessed using Egger [36] and Begg [37] tests, and* P* value less than 0.05 was considered to have a significant publication bias. All statistical analyses were performed using the STATA version 14.0 and Meta-DiSc version 1.4. Except where otherwise specified, a* P* value <0·05 was considered to be statistically significant.

## 3. Results

### 3.1. Literature Search

**Figure 1:**
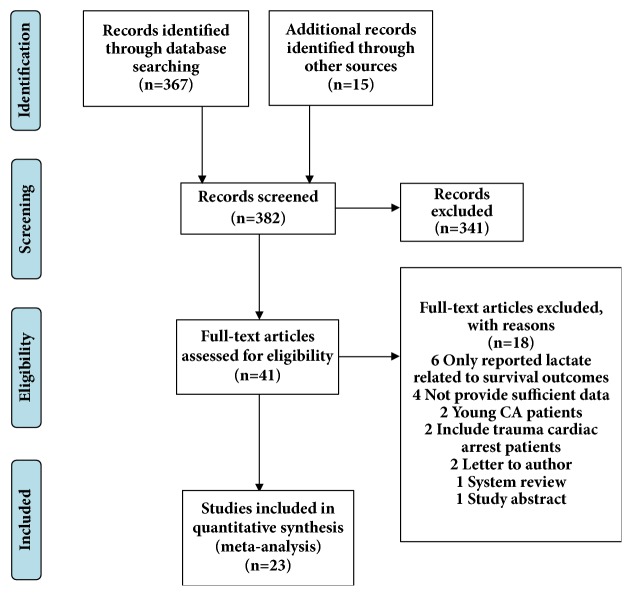
Flow diagram of study selection process.

The systematic literature search yielded 382 potentially relevant records (Figure 1). After excluding duplicates and clearly irrelevant publications by reading titles and abstracts, we obtained 41 full articles of potentially relevant studies for a further evaluation. After full-text reviews, 18 out of 41 articles were excluded. Six articles reported lactate levels related to survival outcome; 4 articles did not provide sufficient data; 2 articles carried on CA patients under the age of 18; 2 articles included trauma cardiac arrest patients; 2 articles were letter to author; 1 was system review; and 1 was study abstract. Finally, 23 articles fulfilled our inclusion criteria [3, 4, 10, 12–31].

### 3.2. Study Characteristics

Baseline information of included studies was provided in Table 1. The included 23 studies contained data from 6720 CA patients. 14 studies were retrospective design. Studies were conducted in countries including Japan (n=7), Korea (n=5), USA (n=4), Australia (n=2), France (n=2), Belgium (n=1), Finland (n=1), and Norway (n=1). The location of CA of patients includes OHCA (n=19) and IHCA/OHCA (n=4). The mean age of patients ranged from to 51 to 78. The outcomes measuring time ranged from discharge from hospital (DC) to 6 months. Quality scores based on NOS scale ranged from 4 to 8. 14 studies were defined as high quality (Table S1).

### 3.3. Lactate Level and Neurological Outcomes

**Figure 2:**
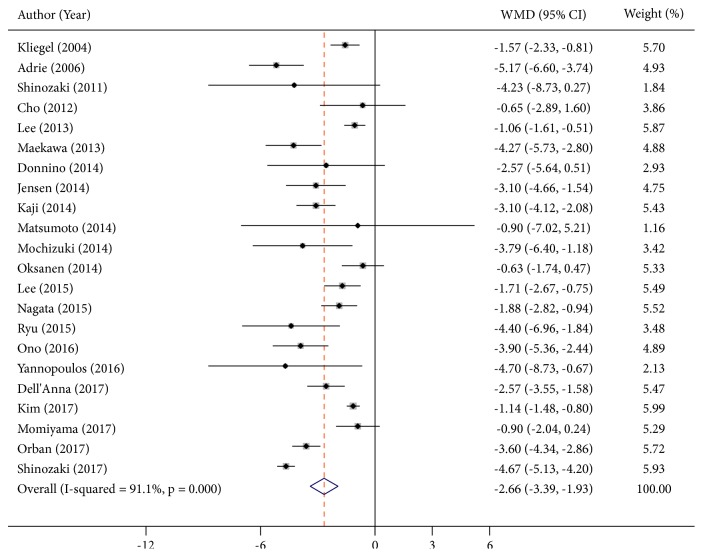
Summary weighed mean difference (WMD) and 95% confidence intervals for lactate on admission between good and poor neurological outcomes (mmol/L).

**Figure 3:**
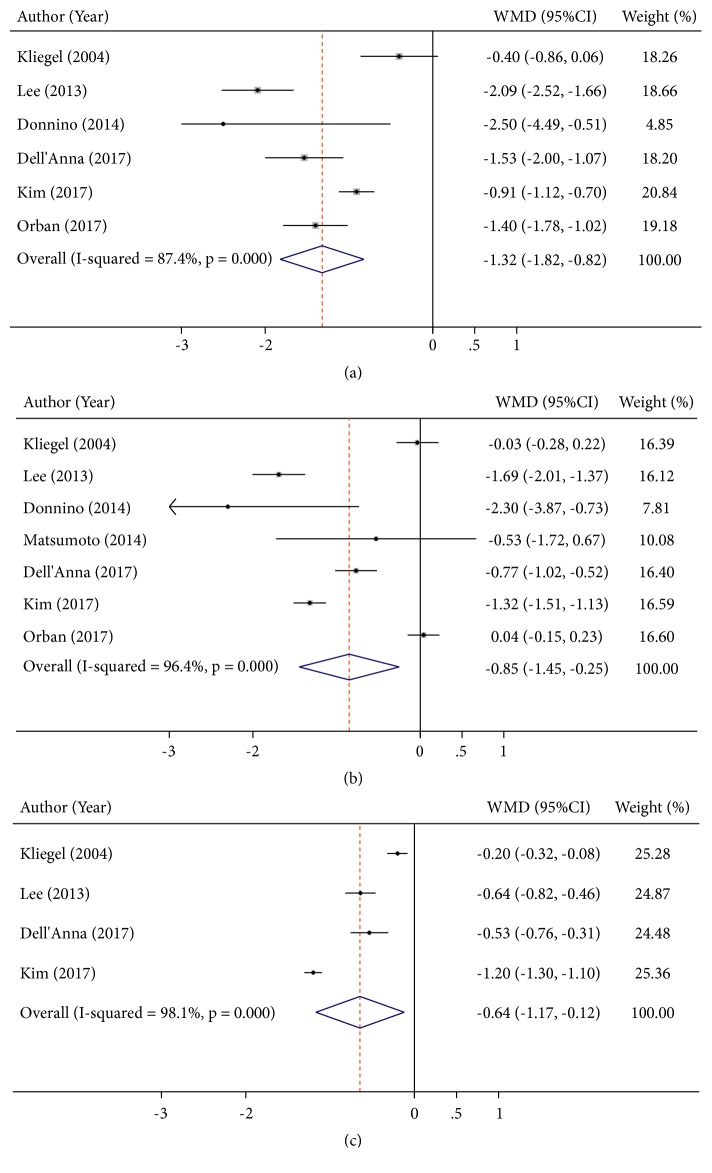
Summary weighed mean difference (WMD) and 95% confidence intervals for lactate at 12h (a), 24h (b), and 48h (c) between good and poor neurological outcomes (mmol/L).

22 articles [3, 4, 10, 13–31] reported lactate levels at different time points related to neurological outcomes, which contained 6,553 participants. These studies were highly heterogeneous within I^2^ > 90%. Consequently, the random effect model was utilized to combine effect size. The WMD on admission was -2.66 mmol/L (95%CI: -3.39 to -1.93,* P*<0.001, I^2^ = 91.1%) (Figure 2). More importantly, a significant result was also found at other time points. The WMD for 12h after admission was -1.32 mmol/L (95%CI: -1.82 to -0.82,* P*<0.001, I^2^ = 87.4). The WMD for 24h after admission was -0.85 mmol/L (95%CI: -1.45 to -0.25,* P*=0.005, I^2^ = 96.4). The WMD for 48h after admission was -0.64 mmol/L (95%CI: -1.17 to -0.12* P*=0.016, I^2^ = 98.1) (Figure 3).

**Figure 4:**
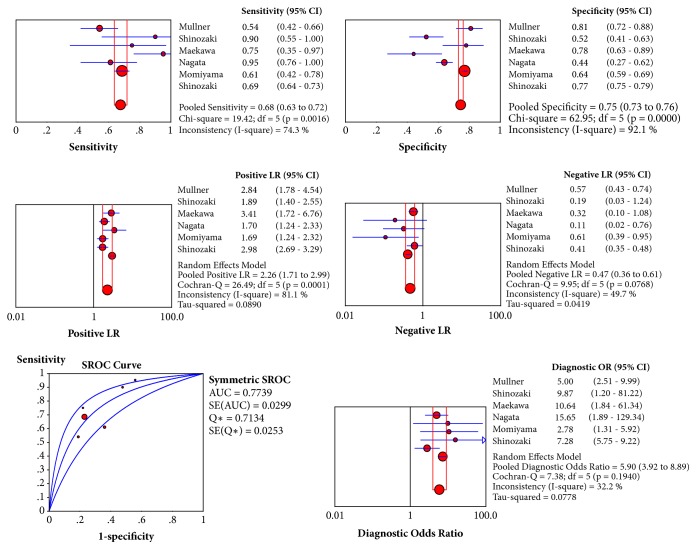
Pooled sensitivity, specificity, positive likelihood ratio (PLR), negative likelihood ratio (NLR), receiver operating characteristic curves (SROC), and diagnostic OR of serum lactate on admission for differentiating neurological outcomes after cardiac arrest.

In addition, we also pooled date of diagnostic test to evaluate the diagnostic efficiency of lactate level on admission. Nine studies reported the date of predict diagnostic test [12, 15, 17, 22, 23, 25, 27, 29, 31] and the information was shown in Table S2. But only six studies reported sufficient data to calculate the number of true positive (TP), false positive (FP), false negative (FN), and true negative (TN) [12, 15, 17, 23, 29, 31]. The Spearman correlation coefficient was 0.771 (*P* = 0.072), suggesting that there was no threshold effect. The pooled sensitivity was 69% (95%CI: 64%-73%); specificity was 77% (95%CI: 75%-79%); positive likelihood ratio was 2.98 (95%CI: 2.69-3.29); negative likelihood ratio was 0.41 (95%CI: 0.35-0.48); and diagnostic odds ratios (DOR) was 5.90 (95%CI: 3.92-8.89). Receiver operating characteristic curves (SROC) did not show a curve in the top left corner of the plot which further indicated the lack of threshold effect and AUC of SROC curve was 0.77 (95%CI: 0.73-0.80) which indicated a moderate diagnostic efficiency of lactate level on admission to predict neurological outcomes after CA. (Figure 4).

Due to limited studies in other time points, the subgroup analysis was only conducted on admission. Subgroup analysis was conducted based on mean age of CA patients, CA location outcome follow-up time, and study design. Age of patients, CA location or outcomes follow-up time did not substantially invert the difference of admission lactate level between good, and poor neurological outcomes or change the heterogeneity between studies (Table 2), whereas when the studies were stratified by study design, heterogeneity was vanished in studies with a prospective design (WMD: -4.47 mmol/L, 95%CI: -4.86 to -4.08,* P*=0.432, I^2^ = 0.2%). In addition, the results of metaregression analysis showed that lactate level on admission would be another important heterogeneity source (*P*=0.049<0.05) (Figure.  S1).

### 3.4. Lactate Clearance and Neurological Outcome

**Figure 5:**
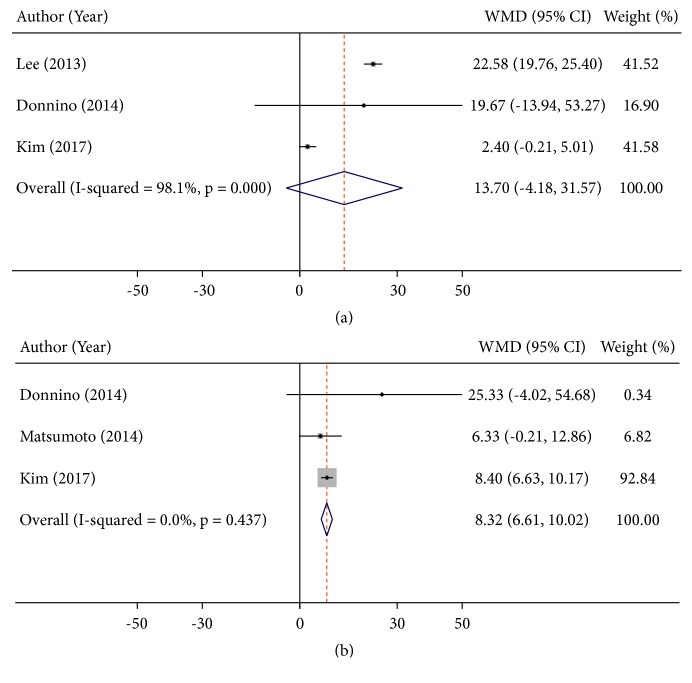
Summary weighed mean difference (WMD) and 95% confidence intervals for lactate clearance rate at 12h (a) and 24h (b) after admission between good and poor neurological outcomes.

Four studies measured lactate clearance related to neurological outcomes [3, 4, 10, 28]. Three studies reported 12h and 24h lactate clearance of patients with different neurological outcomes [3, 4, 28] and the remaining study only reported 24h lactate clearance of patients between good and poor neurological outcomes patients [10]. A significant higher 24h lactate clearance was found in patients with good neurological outcomes (WMD: 8.32%, 95%CI: 6.61 to 10.02,* P*<0.001, I^2^ = 0.0%), but there was no significant difference of 12h lactate clearance between different neurological outcomes (WMD: 13.70%, 95%CI: -4.18 to 31.57,* P*<0.001, I^2^ = 98.1%) (Figure 5).

### 3.5. Sensitivity and Publication Bias Analysis

In sensitivity analysis, there was not one study substantiality that subverted the WMD of lactate between different neurological outcomes except lactate level at 48h (Figure S2). The funnel plots showed no obvious dissymmetry in the shape of funnel plots in both time points (Figure S3). The* P* value of Begg rank correlation test and Egger liner regression test for lactate on admission was 0.463 and 0.346, respectively. In other time points, the results of Begg and Egger test also suggested no evidence of publication bias (*P *> 0.05).

## 4. Discussion

To the best of our knowledge, this was the first meta-analysis paying a close attention to the association and prognostic value of blood lactate and lactate clearance on CA neurological outcomes through systematically reviewing 23 relevant studies. We found a significant relationship between lactate levels on admission and 12h, 24h, and 24h after admission were associated with neurological outcomes. Particularly lactate level on admission has the diagnostic ability to predict neurological outcomes after CA. But a significant association between lactate clearance and neurological outcomes was only found in 24h but not 12h.

As an organ dysfunction metabolite, lactate level is related to prehospital care factors such as quality of bystander CPR, the duration of down time [38], and initial rhythm and post-CA care factors such as hypothermia therapy. Therefore lactate was measured routinely in cardiac arrest patients or even other critically ill patients to assess short or long-term prognosis [39]. In cardiac arrest patients, patients with a lower lactate level on admission have higher possibility of ROSC [40]. One recent meta-analysis by Debaty et al. [41] also revealed that patients with a lower serum lactate level on hospital admission were associated with better survival outcomes. In our case, we further explored the association between lactate or lactated clearance and neurological outcomes. And we successfully demonstrated the associations between lactate levels on admission and 12h, 24h, and 48h after admission and CA neurological outcomes. Additionally, we also found a robust prognostic value of lactate level on admission to predict neurological outcomes. But if we wanted to apply it as gold standard in post-CA care, it was necessary to combine lactate level with other markers (e.g., blood ammonia, vasopressor) [15, 42].

Studies reported that serum lactate level dramatically decreased within early time after admission (0-6h) but then slowly decreased [3, 4, 28]. A prospective designed multicenter study with 543 OHCA patients also documented that, despite the initial lactate level, effective lactate reduction over the first 6h of postcardiac arrest care was associated with survival and good neurologic outcomes [43]. It was the early lactate clearance which dominated the decrease of serum lactate. In our study, there was no significant difference in 12h lactate clearance rate between good and poor neurological outcomes patients. This was just in accord with the property of lactate metabolism kinetics. On the other hand, even we obtained a positive result in the association between 24h lactate clearance and neurological outcomes, but 2 out of 3 included studies reporting negative results. The remaining one study reported that the association between lactate clearance and neurological outcomes disappeared at 12h and 24h after adjusting for potential confounders [28]. In summary, lactate clearance was not robust to predict neurological outcomes after CA.

Unique to recent meta-analysis which focuses on the lactate level on hospital admission and CA survival and neurological outcomes [41], we included more studies into our analysis, so the results were comprehensive and reliable. Another strength was that we conducted a summary of prognostic test to evaluate the neurological outcomes prognostic efficiency of lactate level on admission. Finally, we also explored the association between lactate levels after admission and lactate clearance related to neurological outcomes. This was exactly another unique advantage.

However, there still existed some limitations in our studies. Firstly, the heterogeneity between studies was obviously due to the huge discrepancy existing between studies, which also created some instability in our results to some extent. But these heterogeneities largely attributed to studies design as we found in our subgroup analysis. The bias of retrospective design in studies may contribute to the obvious heterogeneity in the analysis of lactate on admission and neurological outcomes. In addition to the study design, another important heterogeneity source we found in metaregression was the lactate level on admission. These two heterogeneity sources may contribute to not only the obvious heterogeneity existing in the analysis of lactate level on admission and neurological outcomes but also other analyses.

Secondly, even though we defined the association between lactate level on admission and CA neurological outcomes on OHCA and IHCA/OHCA, we did not confirm it in IHCA patients. Dell'Anna et al. found that there was no significant difference of lactate level between IHCA and OHCA [27]. Another system review obtained the result that lactate level on admission was associated with CA outcomes in IHCA patients treated with extracorporeal cardiopulmonary resuscitation [44], even though there was still huge heterogeneity between IHCA and OHCA population. Thirdly, the definition of lactate measuring time points was differed between studies. It was hard to make a uniform definition of lactate measured time points. Thus, it would be more reasonable to interpret with the time points in our study as a rough time division. Finally, the number of studies was limited in the analysis of lactate clearance and neurological outcomes which would also influence the stability of the results.

In conclusion, this meta-analysis took an overview of the relationship of lactate level and lactate clearance and CA neurological outcomes. We found that lactate level on admission and 12h, 24h, and 48h after admission was significantly associated with neurological outcomes. Additionally, lactate level on admission had a moderate neurological outcomes prognostic value. But, the lactate clearance was not robust to predict neurological outcomes after CA. Lactate level was more robust than lactate clearance to predict neurological outcomes after CA. But in view of the heterogeneity between studies, these findings should be interpreted with cautions and still need more large population, prospective design, and clinical studies with superior quality control to verify.

## Figures and Tables

**Table 1:** Characteristics of included clinical studies.

Studies	Year	Design	Country	Age (mean)	Sex, M/F	Location of CA	Percentage of initial shockable rhythm	No.	Outcome follow up	Hypothermia (%)	Measure from	Neurological outcomes (G/P)	Lactate level on admission (G/P) (mmol/L)
Mullner [12]	1997	Retrospective	Australia	62	152/15	OHCA	VF (100%)	167	6 m	None	Arterial	73/94	-
Kliegel [13]	2004	Retrospective	Australia	58	282/112	IHCA/OHCA	VF/VT (65.5%)	394	6 m	None	Arterial	186/208	7.77/9.33
Adrie [14]	2006	Prospective	France	57	95/35	OHCA	VT/VF (42%)	130	DC	11	None	28/102	3.27/8.43
Shinozaki [15]	2011	Prospective	Japan	68	63/35	OHCA	VF/VT (14.3%)	98	6 m	20	None	10/88	7.61/11.84
Cho [16]	2012	Retrospective	Korea	51.3	82/25	OHCA	VF/VT (29.9%)	117	1 m	100	None	34/83	8.89/9.53
Lee [3]	2013	Retrospective	Korea	51.7	48/28	OHCA	VF (43.4%)	76	1 m	100	Venous	34/42	6.07/7.13
Maekawa [17]	2013	Prospective	Japan	54	44/8	OHCA	VF/VT (59.6%)	52	3 m	50	Arterial	8/44	12.70/16.97
Donnino [4]	2014	Prospective	USA	63	60/40	OHCA	VF/VT (55%)	100	DC	97	None	30/16	4.23/6.80
Jensen [18]	2014	Prospective	Norway	59.2	87/24	OHCA	VF/VT (79.3%)	111	DC	100	None	53/58	6.50/9.60
Kaji [19]	2014	Retrospective	USA	65	98/86	OHCA	VT/VF (35.9%)	184	DC	63.2	None	43/141	3.47/6.57
Matsumoto [10]	2014	Prospective	Japan	49.1	11/2	OHCA	VF (100%)	13	1 m	100	Arterial	7/6	12.25/13.15
Mochizuki [20]	2014	Retrospective	Japan	51	33/17	OHCA	VF/VT (54%)	50	1 m	32	None	10/40	10.94/14.73
Oksanen [21]	2014	Retrospective	Finland	58.6	89/20	OHCA	VF (100%)	109	DC	100	None	81/28	3.00/3.63
Lee [22]	2015	Retrospective	Korea	57.7	295/148	OHCA	VF (22.6%)	443	DC	100	None	96/347	8.99/10.70
Nagata [23]	2015	Retrospective	Japan	76.8	39/16	OHCA	VT/VF (45.5%)	55	DC	45.5	None	21/34	3.23/5.11
Ryu [24]	2015	Retrospective	Korea	58	80/35	IHCA/OHCA	VT/VF (42%)	115	DC	8.0	None	68/47	7.87/12.27
Ono [25]	2016	Prospective	Japan	70.5	180/135	OHCA	VT/VF (24.1%)	315	1 m	30.2	None	51/264	5.37/9.27
Yannopoulos [26]	2016	Prospective	USA	18-75	14/4	OHCA	VF (100%)	18	1 m	100	None	9/9	9.90/14.60
Dell'Anna [27]	2017	Retrospective	Belgium	63	155/81	IHCA/OHCA	VT/VF (42%)	236	3 m	100	Arterial	74/162	3.17/5.73
Kim [28]	2017	Retrospective	Korea	60.5	183/99	IHCA/OHCA	VT/VF (35.5%)	282	DC	100	Arterial	98/184	6.46/7.6
Momiyama [29]	2017	Retrospective	Japan	73	236/136	OHCA	VT/VF (89%)	372	DC	100	Arterial	31/341	5.26/6.15
Orban [30]	2017	Retrospective	France	65	192/80	OHCA	VT/VF (44%)	272	DC	100	Arterial	89/183	2.43/6.03
Shinozaki [31]	2017	Prospective	USA	70.8	1772/973	OHCA	VT/VF (42%)	3011	3 m	None	None	380/2365	7.03/11.70

CA: cardiac arrest; DC: discharge from hospital; G: good neurological outcomes; IHCA: in hospital CA; N: no information; m: month; OHCA: out of hospital CA; P: poor neurological outcomes; VF: ventricular fibrillation; VT: ventricular tachycardia.

**Table 2:** Subgroup analysis of lactate on admission and neurological outcomes.

**Subgroups**	**No. of study**	**WMD**	**95**%**CI**	***P* for heterogeneity**	**I** ^**2**^ ** (**%**)**
**Age**					
Adult	14	-2.27	-2.94, -1.69	0.000	80.2
Old people	8	-3.17	-4.26, -1.60	0.000	87.5
**CA Location**					
OHCA	18	-2.79	-3.65, -1.93	0.000	89.5
OHCA/IHCA	4	-1.93	-2.82, -1.04	0.004	77.1
**Outcome follow up time**					
Short-term (< 1m)	18	-2.52	-3.20, -1.84	0.000	84.5
Long-term (≥ 3 m)	4	-3.10	-5.04, -1.16	0.000	94.2
**Study quality**					
Intermediate (≤6)	8	-2.88	-4.12, -1.64	0.000	77.2
High (>6)	14	-2.56	-3.48, -1.64	0.000	93.6
**Data transformation**					
Yes	15	-3.00	-3.84, -2.16	0.000	86.4
No	7	-1.53	-2.09, -0.97	0.03	56.9
**Initial shockable rhythm **					
Low percentage (<50%)	13	-2.83	-3.80, -1.87	0.000	94.2
High percentage (≥50%)	9	-2.28	-3.28, -1.29	0.001	68.1
**Study design**					
Retrospective	13	-1.92	-2.50, -1.35	0.000	81.2
Prospective	9	-4.47	-4.86, -4.08	0.432	**0.2**

## Data Availability

All original data used to support the findings of this study are available from the corresponding author upon request.

## Supplementary Materials

Supplementary Materials Figure S1: metaregression plots of the association between mean changes in lactate concentrations after cardiac arrest and some baseline characteristics. Figure S2: sensitive analysis plots at different time points. Figure S3: funnel plots of included studies at different time points. Table S1: methodological quality assessment of included studies by Newcastle-Ottawa Scale. Table S2: the ability of lactate on admission to predict neurological outcomes.
