# Supplementary material for: Blood Lactate or Lactate Clearance: Which Is Robust to Predict the Neurological Outcomes after Cardiac Arrest? A Systematic Review and Meta-Analysis
Source: Biomed Res Int. 2018 Oct 2;2018:8014213. doi: 10.1155/2018/8014213 (PMC6189651; doi:10.1155/2018/8014213)
Supplement: Supplementary Materials — Figure S1: metaregression plots of the association between mean changes in lactate concentrations after cardiac arrest and some baseline characteristics. Figure S2: sensitive analysis plots at different time points. Figure S3: funnel plots of included studies at different time points. Table S1: methodological quality assessment of included studies by Newcastle-Ottawa Scale. Table S2: the ability of lactate on admission to predict neurological outcomes. [file 8014213.f1.docx]

**
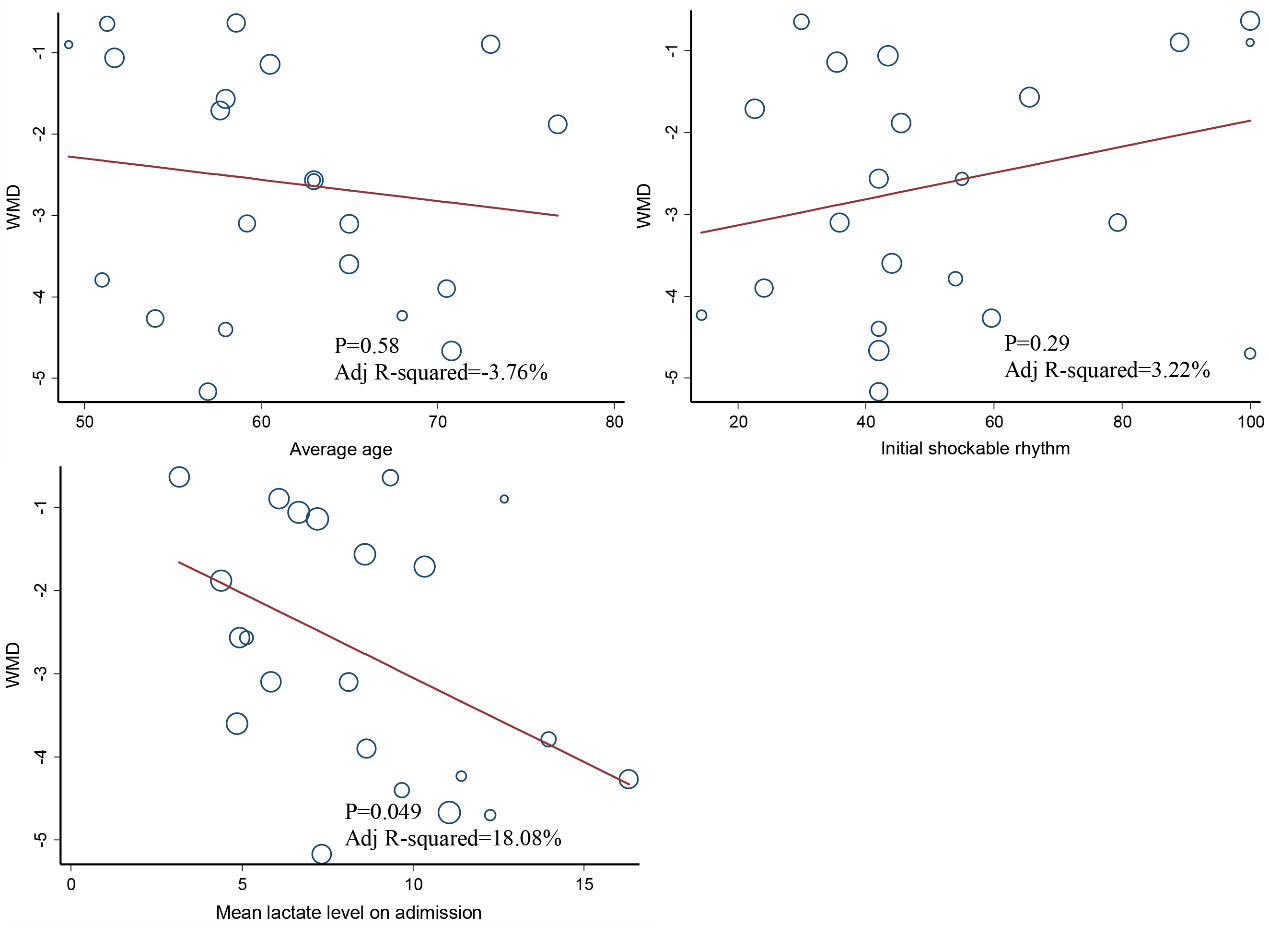
**

**Figure. S1** Meta-regression plots of the association between mean changes in lactate concentrations after cardiac arrest with average age, initial shockable rhythm and mean lactate level on admission.

**(a)**

**(b)**

**(c)**

**(d)**

**Figure. S2** Sensitive analysis plots of summary weighed mean difference (WMD) and 95% confidence intervals for lactate on admission (a) and 12h (b), 24h (c) and 48h (d) after admission between good neurological and poor neurological outcomes (mmol/L).

**(a)**

**(b)**

**(c)**

**(d)**

**Figure. S3** Funnel plots for testing publication bias at different time points: (a) admission; (b) 12h after admission; (c) 24h after admission (d) 48h after admission.

**Table. S1 Methodological quality assessment of included studies by** **Newcastle-Ottawa Scale.**

| **Study** | **Selection** | | | | **Comparability** | **Outcome** | | | **Total score** |
| --- | --- | --- | --- | --- | --- | --- | --- | --- | --- |
|  | **Representativeness of the exposed cohort** | **Selection of the non-exposed cohort** | **Ascertainment of exposure** | **Demonstration that outcome of interest was not present at start of study** | **Comparability domain** | **Assessment of outcome** | **follow-up long enough for outcomes to occur** | **Adequacy of follow up of cohorts** |  |
| Mullner, 1997 | 1 | 1 | 1 | 1 | 0 | 0 | 1 | 0 | 6` |
| Kliegel, 2004 | 1 | 1 | 1 | 1 | 1 | 0 | 1 | 1 | 7 |
| Adrie, 2006 | 1 | 1 | 1 | 1 | 1 | 1 | 0 | 0 | 6 |
| Shinozaki, 2011 | 0 | 1 | 1 | 1 | 1 | 0 | 1 | 1 | 7 |
| Cho, 2012 | 1 | 1 | 1 | 1 | 1 | 1 | 1 | 0 | 7 |
| Lee,2013 | 1 | 1 | 1 | 1 | 1 | 0 | 1 | 1 | 7 |
| Maekawa, 2013 | 1 | 1 | 1 | 1 | 1 | 0 | 1 | 1 | 7 |
| Donnino, 2014 | 1 | 1 | 1 | 1 | 1 | 1 | 0 | 0 | 6 |
| Jensen, 2014 | 1 | 1 | 1 | 1 | 1 | 1 | 0 | 1 | 7 |
| Kaji, 2014 | 1 | 1 | 1 | 1 | 1 | 1 | 0 | 0 | 6 |
| Matsumoto, 2014 | 0 | 1 | 1 | 1 | 0 | 0 | 1 | 0 | 4 |
| Mochizuki, 2014 | 1 | 1 | 1 | 1 | 0 | 0 | 1 | 1 | 6 |
| Oksanen, 2014 | 0 | 1 | 1 | 1 | 1 | 1 | 0 | 1 | 6 |
| Lee, 2015 | 1 | 1 | 1 | 1 | 1 | 1 | 0 | 1 | 7 |
| Nagata, 2015 | 0 | 1 | 1 | 1 | 1 | 1 | 0 | 0 | 5 |
| Ryu, 2015 | 0 | 1 | 1 | 1 | 1 | 1 | 0 | 1 | 6 |
| Ono, 2016 | 1 | 1 | 1 | 1 | 1 | 1 | 1 | 0 | 7 |
| Yannopoulos, 2016 | 1 | 1 | 1 | 1 | 1 | 0 | 1 | 1 | 7 |
| Dell’Anna, 2017 | 1 | 1 | 1 | 1 | 1 | 1 | 1 | 1 | 7 |
| Kim, 2017 | 1 | 1 | 1 | 1 | 1 | 1 | 0 | 1 | 7 |
| Momiyama, 2017 | 1 | 1 | 1 | 1 | 1 | 1 | 0 | 1 | 7 |
| Orban, 2017 | 1 | 1 | 1 | 1 | 1 | 1 | 0 | 1 | 7 |
| Shinozaki, 2017 | 1 | 1 | 1 | 1 | 1 | 1 | 1 | 1 | 8 |

**Table. S2 The ability of lactate on admission to predict neurological outcomes**

| **Author/Year** | **Lactate cut-off point (mmol/l)** | **Measure time** | **Outcome measure time** | **N** | **Sensitivity (%)** | **Specificity (%)** | **False positive (%)** | **False negative (%)** | **AUC** | **95% CI** |
| --- | --- | --- | --- | --- | --- | --- | --- | --- | --- | --- |
| Mullner, 1997 | 10.3 | admission | 6 m | 167 | 54 | 81 | 19 | 46 | - | - |
| Shinozaki, 2011 | 12 | admission | DC | 98 | 90 | 52.3 | 47.7 | 10 | 0.735 | 0.57-0.90 |
| Maekawa, 2013 | 13 | admission | 3 m | 52 | 75 | 78 | 22 | 25 | 0.73 | 0.57-0.9 |
| Lee, 2015 | 7.71 | admission | DC | 443 | - | - | - | - | 0.614 | 0.55-0.68 |
| Nagata, 2015 | 8.86 | admission | DC | 55 | 95.2 | 44.1 | 55.9 | 4.8 | 0.82 | 0.7-0.88 |
| Ono, 2016 | 6.7 | admission | 1 m | 315 | - | - | - | - | 0.726 | 0.64-0.81 |
| Dell'Anna, 2017 | - | admission | 3 m | 236 | - | - | - | - | 0.69 | 0.62-0.75 |
| Momiyama, 2017 | 7.05 | admission | DC | 372 | 61 | 64 | 36 | 39 | 0.62 | 0.51-0.74 |
| Shinozaki, 2017 | 8.5 | admission | DC | 3011 | 68.5 | 77 | 23 | 31.5 | 0.62 | 0.51-0.74 |
| AUC: area under the curve; CA: cardiac arrest; DC: discharge from hospital; m: month | | | | | | | | | | |
